# A care substitution service in the Netherlands: impact on referral, cost, and patient satisfaction

**DOI:** 10.1186/s12875-023-02137-y

**Published:** 2023-09-01

**Authors:** Trijntje Albada, Marjolein Y. Berger, Wim Brunninkhuis, Daphne van Kalken, Karin M. Vermeulen, Robert J. Damstra, Gea A. Holtman

**Affiliations:** 1 Department of Primary and Long-term Care, University of Groningen, University Medical Center Groningen, PO Box 196, 9700 AD Groningen, the Netherlands; 2 Project Group Care Substitution Service Regiopoli Sunenz Drachten, Drachten, the Netherlands; 3 Department of Epidemiology, University of Groningen, University Medical Center Groningen, Groningen, the Netherlands; 4 Department of Dermatology, Phlebology and Lympho-vascular Medicine, Nij Smellinghe Hospital, Drachten, the Netherlands

**Keywords:** Primary health care, Referral and consultation, Right care at the right place, Substitution

## Abstract

**Background:**

In care substitution services, medical specialists offer brief consultations to provide general practitioners (GPs) with advice on diagnosis, treatment, or hospital referral. When GPs serve as gatekeepers to secondary care, these regional services could reduce pressures on healthcare systems. The aim is to determine the impact of implementing a care substitution service for dermatology, orthopaedics, and cardiology on the hospital referral rate, health care costs, and patient satisfaction.

**Methods:**

A before-after study was used to evaluate hospital referral rates and health care costs during a follow-up period of 1 year. The study population comprised patients with eligible International Classification of Primary Care codes for referral to the care substitution service (only dermatology, orthopaedic, cardiology indications), as pre-defined by GPs and medical specialists. We compared referral rates before and after implementation by χ^2^ tests and evaluated patient preference by qualitative analysis.

**Results:**

In total, 4,930 patients were included, 2,408 before and 2,522 after implementation. The care substitution service decreased hospital referrals during the follow-up period from 15 to 11%. The referral rate decreased most for dermatology (from 15 to 9%), resulting in a cost reduction of €10.59 per patient, while the other two specialisms experienced smaller reductions in referral rates. Patients reported being satisfied, mainly because of the null cost, improved organisation, improved care, and positive experience of the consultation.

**Conclusions:**

The care substitution service showed promise for specialisms that require fewer hospital facilities, as exemplified by dermatology.

**Supplementary Information:**

The online version contains supplementary material available at 10.1186/s12875-023-02137-y.

## Background

Population ageing, greater disease chronicity, and technological advancements have placed increasing pressures on healthcare systems worldwide [1]. The International Monetary Fund has stated that spending on health care is a key driver of total domestic spending and predict further increases over time [2–4]. Countries must therefore develop more sustainable healthcare systems, with care substitution being developed to provide healthcare at the right time and place while maintaining quality [5]. A recently published review identified that care substitution in general practice involves either specialist consultation in a primary care setting or joint consultation by a general practitioner (GP) and a medical specialist [6]. These services typically resulted in higher patient satisfaction, shorter clinic waiting times, and shorter waiting lists. The Dutch Ministry of Health has nominated several regions to experiment with care substitution. A service implemented in the northern part of the Netherlands offers specialist consultations for dermatology [7], orthopaedics, and cardiology in a local health centre. However, the effects of this service on hospital referrals and costs are unknown, and as a new intervention, it requires evaluation in this dynamic and region-specific context.

The current study aims to evaluate the impact of a Dutch care substitution service on hospital referral rates including economic evaluation and qualitative analysis of patient satisfaction.

## Methods

### Study design and setting

We performed a study comparing referral rates from primary care before (1-1-2014 to 1-1-2015) and after (15-5-2016 to 15-5-2017) implementing a care substitution service. Impact on referral rates and costs was evaluated after 12 months follow-up from first consultation. In addition, a qualitative analysis of patient satisfaction was performed directly after the care substitution service consultation. The care substitution service (named ‘Regiopoli’) was established in January 2016 for dermatology, orthopaedic, and cardiology consultations in a local health centre. It runs in the north of the Netherlands as a partnership between a GP co-operative, various medical specialists, the regional hospital, a local health centre, and a health insurance company. The region has 120,000 residents served by one hospital and approximately 90 GPs within 25 km of the Regiopoli.

### Procedure

#### Enrolment

All regional GPs were invited to participate in the study and we considered the GPs practice distribution across the area when including the GPs. Participating GPs were asked to provide routine health care data of patients fulfilling the inclusion criteria. Patients who visited a participating GP in the study period with a new episode based on the pre-defined International Classification of Primary Care (ICPC) list were included. A patient could participate only once per ICPC episode, but they could be included multiple times if they presented with other pre-defined ICPCs (counted as new patients). We excluded prevalent cases (i.e., consulting for an ongoing episode or already receiving specialist treatment as these were not eligible for referral to Regiopoli), patients aged <18 years, and cases requiring acute hospital referral.

#### The care substitution service

GPs coded patient contact for presentations (episodes) according to ICPCs for suitable referral indications. The list was based on the Dutch college of GPs guidelines and the ICPCs were agreed in a consensus meeting between 2 GPs, 2 dermatologists, 1 orthopaedic, and 2 cardiologists (Table 1). When uncertain about hospital referral for an approved ICPC indication, a GP could refer their patient for care substitution by a specialist (4 dermatologists, 1 orthopaedic surgeon, and 2 cardiologists). After a short 1:1 consultation with the specialist at the health centre, the specialist provided advice to the GP on diagnosis, treatment, or referral. The GP retained responsibility for patients throughout. A dedicated electronic patient file (HIX, Chipsoft®) connected to an integrated referral system (Zorgdomein®) facilitated automatic encrypted communication with the GP system (Medicom®). Patients did not contribute to the cost of this care, contrasting with usual hospital care in the Netherlands (max €385.- in 2016/2017). Consultation rooms were not equipped with additional test facilities, though cardiology had access to electrocardiography.

**Table 1:** The ICPC codes selected as indications for referral to the care substitution service

**ICPC codes**	
**Orthopaedics**	
**L03**	Low back pain without radiation
**L15**	Knee symptoms
**L83**	Hernia spine
**L84**	Arthrosis
**L92**	Shoulder complaints
**L93**	epicondylitis
**L98**	Twisted abnormality extremities
**L99**	Other musculoskeletal disease(s)
**Cardiology**	
**K77**	Congestive heart failure
**K78**	Atrial fibrillation
**K81**	Heart murmur
**Dermatology**	
**S04**	Local swelling, papule, nodule skin, subcutis
**S05**	Multiple swellings/papules/lumps of skin/subcutis
**S77**	Skin malignancy
**S79**	Other benign neoplasm skin
**S87**	Constitutional eczema
**S82**	Nevus, birthmark
**S88**	Contact eczema, other eczema
**S96**	Acne
**S99**	Other sin diseases, subcutis

*ICPC* International Classification of Primary Care

#### Patient satisfaction

After their consultations, the medical specialist asked patients to complete a patient satisfaction questionnaire based on the Consumer Quality Index (CQ-index) for GP care [8]. Open questions asked (1) if patients had a preference for substitution care or hospital care and (2) if they had any comments about their substitution care consult.

### Outcomes

The primary outcome was the referral rate to hospital during the 1-year follow-up period. Secondary outcomes included the time between GP referral and care substitution or hospital consultation, the referral rate to allied healthcare professionals in primary care during the 1-year follow-up period, and the healthcare cost per patient per year. One researcher extracted these outcomes retrospectively from the GPs’ electronic medical record system into a pre-defined anonymised database. In addition, we were interested in patient preference for, and satisfaction with, the care substitution service.

### Analysis

The three specialisms had estimated average referral rates of 14% based on data from Nivel, a Dutch public knowledge organisation that conducts research into healthcare [9]. To detect a targeted 50% decrease in the number of hospital referrals for each specialism with 80% power and a 5% type I error, we required an estimated minimum sample size of 1800 (2 groups × 3 specialisms × 300 patients).

GP, patient, and consultation details are reported descriptively. The referral rate was determined by dividing the total number of referrals by the total number of patients (overall and per specialism) included during the 1-year follow-up period; differences before and after implementation were then evaluated by the chi-square test. We also compared the median number of days between GP referral and specialist consultation in the periods before and after implementation by specialism.

We compared total costs and alternative confidence intervals indicated by bootstrapping (5000 replications) before and after implementation when evaluating costs per patient per year. Total healthcare costs were based on number and cost of consultations and referrals during follow-up. These were estimated using a national database of the Dutch Healthcare Authority and a national database with standardised average costs for hospital care by consultation type [10], set at the 2018 price level. We did not include costs for diagnostics, medication, or social care.

Seven questions on patient satisfaction in the questionnaire were evaluated descriptively, and the two open questions were evaluated thematically. Two authors (TA, GAH) conducted the thematic analysis by data familiarisation (using 10% of the responses), open coding, and inductive reasoning to identify categories within derived themes. The same two researchers then scored all questionnaire data independently in the identified categories, adding new categories when scoring was not possible and discussing the codes and categories afterwards. Finally, key themes for referral preference (substitution or hospital) were developed during a consensus meeting with a third researcher.

## Results

### GP and patient characteristics

Specialists at the care substitution service saw 1190 patients referred by 82 GPs during the post-implementation period. Of these, 7 GPs (5 males, median age 49 [IQR 46–50] years) with 9 (IQR 6–11) years' work experience referred patients and provided routine health care data for 2,408 and 2,522 patients before and after implementation, respectively (Table 2). The distance from their practices to the care substitution service ranged from 2.5 to 24.5 km. All 7 GPs provided data for cardiology, 5 for dermatology, and 4 for orthopaedics. Patients in the two study groups had similar ages and comorbidities, though by specialism, patients seen by cardiology were older and had a co-morbidity more often than those seen by the other two specialisms.

**Table 2:** Baseline characteristics before and after implementing care substitution

	**Before implementation**	**After implementation**
**Total consults, N (%)**		
Overall	2408	2522
Dermatology	1279 (53.11)	1381 (54.76)
Orthopaedics	1072 (44.52)	1087 (43.10)
Cardiology	57 (2.37)	54 (2.14)
**Age in years, mean ± SD**		
Overall	51.18 ± 18.26	53.08 ± 18.06
Dermatology	49.63 ± 18.86	51.94 ± 18.66
Orthopaedics	51.84 ± 16.87	53.48 ± 16.73
Cardiology	73.51 ± 13.96	74.31 ± 14.75
**Female, N (%)**		
Overall	1408 (58.47%)	1465 (58.09%)
Dermatology	772 (60.36%)	816 (59.09%)
Orthopaedics	609 (56.81%)	619 (56.95%)
Cardiology	27 (47.39%)	30 (55.56%)
**Participants with co-morbidity**^**a**^**, N (%)**		
Overall	398 (16.53)	459 (18.20)
Dermatology	174 (13.60)	216 (15.64)
Orthopaedics	205 (19.12)	220 (20.24)
Cardiology	19 (33.33)	23 (42.59)

Before implementation covered 1-1-2014 to 1-1-2015, and after implementation covered 15-5-2016 to 15-5-2017

^a^Comorbidity: diabetes mellitus, stroke, heart failure, cancer, lung disease, relevant joint wear, osteoporosis, relevant bone fracture, depression, anxiety/panic disorder, dementia

### Referrals

**Fig. 1:**
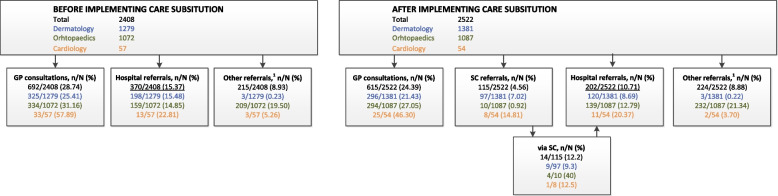
GP consultations and referral rates before and after implementing care substitution ^1^Other referrals: allied healthcare professionals in primary care, namely dietician, occupational therapy, physiotherapy, skin therapy, manual therapy, exercise therapy, podiatry, and home care. GP consultations and referrals are evaluated during the following period: *Before* implementation covered 1-1-2014 to 1-1-2015, and *after* implementation covered 15-5-2016 to 15-5-2017. GP: general practitioner, SC: substitutional care. Note: one episode of one patient counted as one patient (i.e., one episode per ICPC code that started in the study period, with a patient able to have episodes for different ICPC codes)

Figure 1 shows that the overall referral rate to hospital decreased from 15 to 11% after implementing care substitution, resulting in a 27% reduction (*p* < 0.001). Dermatology saw the greatest decrease in hospital referrals, from 15 to 9%, giving a 44% overall reduction (*p* < 0.001). Orthopaedics (15 to 13%; *p* = 0.169) and cardiology (23 to 20%; *p* = 0.760) saw 14% and 11% reductions, respectively.

The dermatologist referred 9 (11%) of the 97 patients they saw to hospital. However, only a few patients were referred to care substitution with orthopaedic (*n* = 10) or cardiovascular (*n* = 8) indications, of which 4 (40%) and 1 (12%) were referred to hospital, respectively. Moreover, the median time from GP referral to specialist consultation was shorter for all patients referred to care substitution (9 days for dermatology, 14 days for orthopaedics, 21.5 days for cardiology).

GPS referred similar numbers to allied healthcare professionals in primary care (Fig. 1) and requested similar numbers of diagnostic tests (Supplementary Table S1) before and after implementation.

### Costs

The mean cost per dermatology patient was €298.92 (95% CI, 244–528) before implementation and €288.32 (95% CI 244–528) after implementation, resulting in an average saving per patient of €10.59. The low referral rates to cardiology and orthopaedics precluded meaningful cost calculations.

### Patient satisfaction 

In total, 711 of 1190 consulting patients (60%) answered the questionnaire: 66% for dermatology, 9% for orthopaedics, 5% for cardiology, and 20% unknown. Of these, 487 (68%) understood the intention of care substitution, 621 (88%) were satisfied after consultation, and 624 (88%) knew the next steps after consultation. Additionally, 298 (51%) of 583 patients appreciated that they did not have to pay a contribution, contrary to the case for a hospital consultation. Care substitution was rated highly overall (8.7/10). Table 3 details the key themes for referral preference, the associated facilitators and barriers, and representative quotes.

**Table 3:** Key themes and associated facilitator and barriers by type of care

**Themes**	**Facilitator/barriers**	**Quotes**
**Preference for substitution care**		
***Financial***	No financial consequences	*‘Don’t have to pay own contribution’*
***Organisation***	Short waiting times between GP visit and substitutional care visit, short waiting times in the waiting room. Good communication between specialist and GP.	*‘Short waiting time, lines are really short’* *‘I experienced a short communication line between specialist and GP’*
***Competent and proper care***	Clear advice and correct/expert care.	*‘Good and clear explanation of the medical problem’*
***Feeling/experience***	Ambience is friendly, attentive, and less clinical. Personal and informal approach. A lot of attention.	*‘Small scale, friendly and very helpful’* *‘Less of an emotional burden than a hospital visit’*
**Preference hospital care**		
***Organisation***	Substitutional care is an unnecessary step, no immediate medicine recipe or follow-up appointment.	*‘First have to go back to my GP, before being referred to hospital’* *‘Immediately receiving medication in the hospital, now it is an unnecessary step’*
***Feeling/experience***	A patient’s feelings.	*‘I like hospital more’* *‘Hospital is specialised’*
**No preference**		
***Competent and proper care***	Quality of care is important.	*‘It’s all about receiving an knowledgeable advice. If such an advice can be provided at the substitutional care it is great, otherwise hospital care is fine’* *‘Receiving proper care is important, place does not matter’*

## Discussion

### Summary

The care substitution service reduced hospital referrals by 27%, but with wide variation among the three specialties. Notably, only dermatology realised a statistically significant decrease, resulting in average cost savings of approximately €10 per patient with a relatively large confidence interval. Waiting times were also shorter for the care substitution service compared to hospital. Patients reported being satisfied with the service, mainly because of the null costs, improved organisation and care, and positive experience of the consultation.

### Strengths and limitations

Practice-based research using routine GP care data is considered a viable alternative to randomised controlled trials, offering valuable connections between science, policy, and practice [11, 12]. It also produces more generalisable data with greater external validity than randomised control trials. Given our expectation of few temporal changes, this design should have limited the disadvantages of the before-after study. In the future, a stepped wedge trial could be used in which clusters are randomised over time, reducing the impact of confounders, especially temporal changes. In addition, despite including many patients eligible for referral, very few were actually referred for orthopaedic and cardiology care substitution and hospital referral rates remained high. The referral criteria for these specialisms need further evaluation.

The low participation of GPs is a major limitation. Nevertheless, despite only including seven GPs, they differed in gender, age, work experience, and distance to the care substitution service; and, by using their practices, we could achieve the required sample size. By evaluating only one care substitution service in one region, it may not be possible to extrapolate the results to other regions, countries, or settings [13]. For example, besides region-specific characteristics, services could have different referral systems. Regiopoli provides a systematic, dedicated, and integrated service within an existing referral system that offers communication between GPs and hospitals in a dedicated electronic record. Nevertheless, our results provide more general insights into the potential facilitators and barriers when implementing such a service.

Our cost evaluation was limited to patient consultations in primary, substitutional, and hospital care settings that used only one DBC. Longitudinal effects should now be investigated at a macro level, considering health care costs from broader medical and societal perspectives, including costs for patient time and travel, medication, and average hospital care DBCs. Nivel have stated, for example, that 2.25 DBCs on average were opened after hospital referral [14], suggesting that the cost for hospital visits in our study are underestimated.

### Comparison with existing literature

Researchers have explored care substitution services in a wide variety of medical specialties [6, 13, 15–17]. Where specialists perform consultations in primary care, these studies have typically shown that care substitution services result in shorter waiting lists, less time in clinics, and higher patient satisfaction than usual care. Our results support the key drivers of satisfaction mentioned in the literature [6, 18]. We found no study with a directly comparable methodology.

#### Dermatology

Smeele et al. [15] assessed hospital referral rates when GPs could refer a patient to substitution care with queries about diagnosis, treatment, or hospital referral. Hospital referral was advised for 21% of patients consulting a dermatologist in that service. By contrast, we used a pre-defined referral protocol and found a referral rate of 9.3%. The difference suggests that using a more defined referral protocol for dermatology may reduce the risk of unnecessary referrals to care substitution services.

#### Orthopaedics

Two out of the five patients (40%) referred to our substitution care orthopedist were additionally referred to hospital. This is comparable to the 44% reported by Smeele et al. [15], who stated that this probably reflected the need for additional hospital facilities and suitably equipped consultation rooms. Another care substitution service with a GP specialist in musculoskeletal disorders instead of an orthopaedist showed that only 4–13% of patients were subsequently referred to hospital via the service [19]. Despite being a descriptive study, the authors concluded that this approach shows promise for orthopaedic care substitution. Future high-quality studies are needed to confirm this hypothesis.

#### Cardiology

The pre-defined referral indications in this study meant that few patients were eligible for cardiology care substitution, and that those referred were older than in the other specialties. An evaluation of care substitution by Quanjel et al. demonstrated that excluding patients with a high probability of hospital referral can improve service efficiency [17]. Gender (male), age (older), referral indicators (stable angina pectoris or dyspnoea), and referral with unclear pathology or to confirm disease increased hospital referrals. Moreover, healthcare costs decreased when they adjusting patient selection according to this profile and excluding patients with a prior cardiology diagnosis or acute problem [18]. However, the reported costs only reflected hospital care provision and excluded primary care, social care, and drug costs. As with the current study, patients preferred substitution care over hospital care [18], citing feeling more welcome and comfortable, being listened to carefully, and receiving more understandable explanations. Another before after study showed that integrating cardiology and primary care improved the follow-up and chronic treatment of patients with ischemic heart disease, heart failure, and atrial fibrillation [20]. They concluded that monitoring of patients was distributed between cardiology and primary care and general practitioners were more satisfied.

### Implications for practice and research

Our results indicate that care substitution services may not offer significant benefits to all specialisms. Dermatology benefitted significantly in this study, possibly because dermatologists rely less on hospital facilities, contrasting with cardiology and orthopaedics that have a greater reliance. However, it is too early to draw any firm conclusions. Efficient and effective care substitution will require better collaboration between GPs and medical specialists to improve referral protocols, taking care to understand the different demands for hospital facilities between specialties, including the differences in patient cohorts, service organisation, and procedures [13]. Usage may improve by engaging senior specialists who share the conviction that care substitution is necessary and by ensuring that GP have positive experiences of care substitution.

The service structure described in this study benefits from its potential to connect more GPs, specialists, and specialties. Indeed, we now expanded the region and have more than 250 referring GPs and new ongoing projects in gynaecology, proctology, otorhinolaryngology. Research should consider the opportunities for other medical specialties, paying attention to the different resources and referral protocols required to achieve effective substitution. In addition, our qualitative research revealed themes that were related to have a preference for substitution care or hospital referral. Future research should now focus on patient preference in greater depth, using semi-structured interviews or focus groups to address these themes. Finally, around 30% did not understood the intention of care substitution, indicating that more information on substitution care for patients is needed.

## Conclusions

This study showed that referral rates were reduced for dermatology but not for orthopaedics or cardiology, possibly implying that care substitution services offer most benefit in specialisms that required fewer specialist facilities. There was little impact on costs. Finally, we showed high rates of satisfaction, but more information about the substitution care could be provided to patients.

### Supplementary Information

**Additional file 1: Table S1. **Overview of diagnostic tests by GPs before and after implementing care substitution.

## Data Availability

The datasets used and/or analysed during the current study are available from the corresponding author on reasonable request.

## Abbreviations

DBC: Diagnose Behandel Combinatie – Diagnosis Treatment Combination
CQ-index: Consumer Quality Index
GP: General practitioners
ICPC: International Classification of Primary Care
IQR: Inter Quartile Range
