# Supplementary material for: A care substitution service in the Netherlands: impact on referral, cost, and patient satisfaction
Source: BMC Prim Care. 2023 Sep 1;24:171. doi: 10.1186/s12875-023-02137-y (PMC10472548; doi:10.1186/s12875-023-02137-y)
Supplement: Supplementary file 1 — Additional file 1: Table S1. Overview of diagnostic tests by GPs before and after implementing care substitution. [file 12875_2023_2137_MOESM1_ESM.docx]

**Table S1.** Overview of diagnostic tests by GPs before and after implementing care substitution

|  | Before implementation | After implementation |
| --- | --- | --- |
| Electrocardiography |  |  |
| Cardiology | 9 | 8 |
| Laboratory |  |  |
| Orthopaedics | 20 | 22 |
| Dermatology | 12 | 14 |
| Cardiology | 17 | 15 |
| Ultrasound |  |  |
| Orthopaedics | 23 | 26 |
| Dermatology | 12 | 10 |
| X-ray |  |  |
| Orthopaedics | 164 | 173 |
| Dermatology | 1 | 1 |
| Cardiology | 1 | 3 |
| Magnetic Resonance Imaging |  |  |
| Orthopaedics | 3 | 1 |
| Computed Tomography |  |  |
| Orthopaedics | 2 | 4 |
| Biopsy |  |  |
| Dermatology | 102 | 108 |
